# Nationwide genetic analysis for molecularly unresolved cystic fibrosis patients in a multiethnic society: implications for preconception carrier screening

**DOI:** 10.1002/mgg3.278

**Published:** 2017-02-19

**Authors:** Doron M. Behar, Ori Inbar, Michal Shteinberg, Michal Gur, Huda Mussaffi, David Shoseyov, Moshe Ashkenazi, Soliman Alkrinawi, Concetta Bormans, Fahed Hakim, Meir Mei‐Zahav, Malena Cohen‐Cymberknoh, Adi Dagan, Dario Prais, Ifat Sarouk, Patrick Stafler, Bat El Bar Aluma, Gidon Akler, Elie Picard, Micha Aviram, Ori Efrati, Galit Livnat, Joseph Rivlin, Lea Bentur, Hannah Blau, Eitan Kerem, Amihood Singer

**Affiliations:** ^1^ Clalit National Personalized Medicine ProgramDepartment of Community Medicine and EpidemiologyCarmel Medical CenterHaifaIsrael; ^2^ Bruce Rappaport Faculty of MedicineTechnion‐Israel Institute of TechnologyHaifaIsrael; ^3^ Gene by GeneGenomic Research CenterHoustonTexas; ^4^ The Cystic Fibrosis Foundation of IsraelRamat GanIsrael; ^5^ Pulmonology Institute and CF CenterCarmel Medical CenterHaifaIsrael; ^6^ Pediatric Pulmonary Institute and CF CenterRappaport Children's HospitalRambam Health Care CampusHaifaIsrael; ^7^ Kathy and Lee Graub Cystic Fibrosis CenterSchneider Children's Medical Center of IsraelPetach TikvaIsrael; ^8^ Sackler Faculty of MedicineTel Aviv UniversityRamat AvivIsrael; ^9^ Cystic Fibrosis CenterHadassah‐Hebrew University Medical CenterJerusalemIsrael; ^10^ Cystic Fibrosis CenterSheba Medical CenterRamat GanIsrael; ^11^ Cystic Fibrosis CenterSoroka Medical CenterBeershevaIsrael; ^12^ Cystic Fibrosis CenterShaare Zedek Medical CenterHebrew University Medical CenterJerusalemIsrael; ^13^ Medical GeneticsBarzilai Medical CenterAshkelonIsrael

**Keywords:** Carrier screening, cystic fibrosis, detection rate, preconception

## Abstract

**Background:**

Preconception carrier screening for cystic fibrosis (CF) is usually performed using ethnically targeted panels of selected mutations. This has been recently challenged by the use of expanded, ethnically indifferent, pan‐population panels. Israel is characterized by genetically heterogeneous populations carrying a wide range of CFTR mutations. To assess the potential of expanding the current Israeli preconception screening program, we sought the subset of molecularly unresolved CF patients listed in the Israeli CF data registry comprising ~650 patients.

**Methods:**

An Israeli nationwide genotyping of 152 CF cases, representing 176 patients lacking molecular diagnosis, was conducted. Molecular analysis included Sanger sequencing for all exons and splice sites, multiplex ligation probe amplification (MLPA), and next‐generation sequencing of the poly‐T/TG tracts.

**Results:**

We identified 54 different mutations, of which only 16 overlapped the 22 mutations included in the Israeli preconception screening program. A total of 29/54 (53.7%) mutations were already listed as CF causing by the CFTR2 database, and only 4/54 (7.4%) were novel. Molecular diagnosis was reached in 78/152 (51.3%) cases. Prenatal diagnosis of 24/78 (30.8%) cases could have been achieved by including all CFTR2‐causing mutations in the Israeli panel.

**Conclusions:**

Our data reveal an overwhelming hidden abundance of *CFTR* gene mutations suggesting that expanded preconception carrier screening might achieve higher preconception detection rates.

## Introduction

Preconception carrier screening of well‐defined deleterious mutations in the CF transmembrane conductance gene (*CFTR*, OMIM #602421), the only gene known to be associated with CF (OMIM #219700), is widely accepted and has been repeatedly advocated for by opinion leaders (American College of Obstetricians and Gynecologists Committee on Genetics, 2011; Castellani et al. 2010; Grody et al. 2013; Langfelder‐Schwind et al. 2014; National Institutes of Health Consensus Development Conference Statement on Genetic Testing for Cystic, 1999; Watson et al. 2004). The reason for this remarkable consensus relates to the life‐threatening nature of the disease and its overall high global frequency mainly among Caucasians (Salvatore et al. 2011). This has led to a plethora of studies suggesting cumulatively over 2000 variants in the *CFTR* gene (http://www.genet.sickkids.on.ca), of which, currently, many have debatable pathogenic effect (Sosnay et al. 2013). Accordingly, the decision on the content of a common mutation panel suitable for population screening programs becomes complex (Watson et al. 2004). Since CF carrier screening programs are not designed to detect all mutations that cause CF, the American College of Medical Genetics (ACMG) has repeatedly revised and suggested panels compatible with population screening of the *CFTR* gene for a pan‐ethnic United States population (Grody et al. 2001; Watson et al. 2004). Other genetic societies have issued guidelines suitable to their respective populations (Delatycki et al. 2014; Wilson et al. 2002). Specifically, the Israeli preconception screening program for CF is comprised of only 22 ethnically targeted mutations (Zlotogora et al. 2016; Zlotogora and Israeli 2009) (Table S1).

Detection rates of preconception CF panels vary considerably depending on the population and the number of screened mutations (Kerem et al. 1995; Lim et al. 2016; Richards et al. 2002). For example, the ACMG recommended United States population panel detection rate is 95–97% among Ashkenazi Jews, but only 57–72% among Hispanic Americans (Grody et al. 2001; Kerem et al. 1995; Quint et al. 2005; Sugarman et al. 2004). Likewise, the detection rate of the Israeli screening program ranges from 97% for Ashkenazi Jews, 92% for Arabs, and reaching 0% for communities such as Ethiopian Jews, and is unknown/low for many other Israeli ethnic groups (Abeliovich et al. 1992; Kerem et al. 1995; Laufer‐Cahana et al. 1999; Quint et al. 2005; Zlotogora et al. 2016). Recently, the pan‐Israeli detection rate obtained by the Israeli screening program was calculated to be 70% (Stafler et al. 2015). This lack of homogeneity in the expected detection rates for the various Israeli subpopulations has been further complicated due to changing demographics secondary to immigration or intercommunity marriages, and the breaking of the link between kindred tribes and their traditional places of residence. In these regard, the American College of Obstetricians and Gynecologists (ACOG) has clearly noted that it is becoming increasingly difficult to assign a single ethnicity to affected individuals and concluded that it is reasonable to offer the same CF carrier screening panel to all individuals regardless of their ethnic background (American College of Obstetricians and Gynecologists Committee on Genetics, 2011). Consistent with the growing discussion regarding expanded preconception carrier screening (Edwards et al. 2015; Grody et al. 2013; Haque et al. 2016; van der Hout et al. 2016), the initial report of the CFTR2 project estimated that testing for 127 variants, meeting both clinical and functional criteria consistent with a disease state would account for 95.4% of CF alleles in their cohort and increase the detection rate for couples undergoing carrier screening from 72% to nearly 91% (Sosnay et al. 2013). More recently, the concern of insufficient detection rates in many populations was assessed, and expanded preconception carrier screening panels for CF have been demonstrated to yield higher detection rates (Lim et al. 2016). Other studies have specifically shown potential benefits for such approaches for diagnosis of CF in newborn screening (Baker et al. 2015; Kammesheidt et al. 2006; Pique et al. 2016).

To study the potential of expanding the currently performed preconception CF screening panel in Israel, we sought the subset of molecularly unresolved CF patients listed in the Israeli CF patient registry (Stafler et al. 2015; Viviani et al. 2014). At the inception of this project, ~450 of the ~650 patients comprising the registry were known to carry two CF‐causing mutations. The remaining ~200 patients were reported to lack a molecular diagnosis despite being previously included in the standard Israeli screening program. The ethnic origins and phenotypes presented by these patients varied significantly. Patients presenting the classical phenotype including severe respiratory disease and pancreatic insufficiency were classified as typical CF, while others demonstrating single organ disease phenotypes, usually with pancreatic sufficiency, were referred to as atypical CF (De Boeck et al. 2006; Wallis 2012). We report the genotyping results of 176 CF patients lacking molecular diagnosis. All patients were subject to the same predefined molecular analysis strategy disregarding their ethnic background. Our major objective was to identify additional CF‐causing mutations among these patients. This may be beneficial for implanting an expanded, global, pan‐population CF preconception screening panel in the multiethnic society of Israel.

## Materials and Methods

### Subjects

All six Israeli CF centers participated in this study. Our goal was to reach out to all patients lacking molecular diagnosis through their respective treating center. A total of 176 patients were enrolled in this study. Written informed consent for genetic testing was given by all the patients or by their parents in the case of minors.

The information collected for each patient included their demographic parameters, gender, age, ethnicity, age of diagnosis, pulmonary status, pancreatic status, liver status, documentation of meconium ileus, sweat chloride values, and any additional clinical relevant information. Based on this information and on commonly practiced criteria, all patients were classified as typical or atypical CF by their treating physicians (De Boeck et al. 2006; Wallis 2012). The clinical diagnosis of CF, or the split of the patients into the typical and atypical phenotypes, was not changed by the molecular data obtained in this study. Males having only congenital absence of the vas deferens (CAVD) were not included.

All CF patients were classified as Jews, Arabs, Druze, or other at the level of all four grandparents. CF Jewish patients were further substratified into their respective ancestral communities. Arab CF patients were substratified as Muslims, Christians, or Bedouin and further according to their residency or tribal ancestry. Parental consanguinity and kinship among participants of the study was documented. Known genealogical relatedness for patients residing in the same village was ascertained by direct questioning.

### Molecular analysis

In most cases, DNA was extracted from buccal swabs and quantitated with a SpectraMax190 (Molecular Devices, Sunnyvale, CA). In a minority of cases, genomic DNA was extracted from peripheral leukocytes following standard protocols. Sanger sequencing of the *CFTR* gene was completed by amplifying the genomic DNA to obtain all *CFTR* gene coding exons and their flanking regions (~20 bp from each side) using conventional PCR techniques. The intronic *CFTR* mutation, c.3717+12191C>T, was also analyzed by direct sequencing. PCR products were purified using magnetic particle technology (Seradyn, Inc., Indianapolis, IN, United States). After purification, all fragments were sequenced in the forward and reverse direction to determine the noted regions. Sequencing was performed on a 3730xl DNA Analyzer (ThermoFisher, Foster City, CA, United States), and the resulting sequences were analyzed with the Sequencher software (Gene Codes Corporation, Ann Arbor, MI, United States). All identified variants were scored relative to the reference sequences deposited in the National Center for Biotechnology Information (CFTR: [NM_000492.3]). *CFTR* chromosomal rearrangements of all exons and intron/exon boundaries were analyzed using MLPA (SALSA MLPA P091 CFTR probemix, MRC‐Holland, Amsterdam, the Netherlands). The poly‐T/TG tracts (hg 19_chr7:117188683‐117188689) were studied by means of NGS techniques. For this purpose, amplicons spanning the region of interest were run on the MiSeq system (Illumina, San Diego, CA) and aligned using NextGene Software (SoftGenetics, State College, PA). The minimum required coverage for each sample was 1000 reads. Genotyping was completed at the Genomic Research Center, Gene by Gene, Houston, TX.

### Mutations classification

The term mutation is used in this manuscript to describe a variant that is pathogenic or likely pathogenic. As the scope of this project did not allow performing functional studies on each of the identified mutations, they were classified based on available information in the literature and the following bioinformatics tools:

1. The current definition of the mutation in CFTR2: CF‐causing mutation, mutation under evaluation, mutation of varying clinical consequence, and non‐CF‐causing mutation (Sosnay et al. 2013).
2. Literature survey of manuscripts previously discussing the mutation.
3. The consequences of the sequence change at the protein level: missense, nonsense, deletion, insertion, duplication, silent, and frame shift.
4. The current definition of the mutation in commonly used databases including dbSNP (Sherry et al. 2001), ClinVar (Landrum et al. 2014), and HGMD (Stenson et al. 2003).
5. Multiple prediction algorithms such as Polyphen (Adzhubei et al. 2010), SIFT (Kumar et al. 2009), and Mutation Taster (Schwarz et al. 2014) were applied on each mutation to assess its potential pathogenicity. Last, REVEL, the recently introduced ensemble method for predicting the pathogenicity of rare missense variants was implemented (Ioannidis et al. 2016).

Following this, each mutation found within this study was categorized as follows:

1. Mutation that affects function:
  - Mutations currently included in the Israeli CF screening program.
  - Mutations currently defined as CF‐causing mutations by CFTR2 (Sosnay et al. 2013).

Mutation that probably affects function:
  - Mutations currently defined as mutations under evaluation or mutations of varying clinical consequences by CFTR2 (Sosnay et al. 2013).
  - Mutations previously reported to be CF‐causing mutations in a peer‐reviewed manuscript including relevant supporting data and not listed in the CFTR2 report (Banjar et al. 1999; Bell et al. 2011; Chillon et al. 1994; Dork et al. 1993; Fanen et al. 1992; Lerer et al. 1999; Mickle et al. 1998; Quint et al. 2005; Romey et al. 1994; Schrijver et al. 2005a; Shoshani et al. 1994; Trujillano et al. 2013).
  - Mutations previously reported to be CAVD‐causing mutations in a peer‐reviewed manuscript(s) including relevant supporting data (Casals et al. 2000; Dork et al. 1997; Schrijver et al. 2005b; Vankeerberghen et al. 1998).
  - Novel deletion mutations creating a frame shift change at the protein level.
  - Novel splice site mutations within the obligatory donor or acceptor sites.
  - Novel missense mutations creating an amino acid change at the protein level which is predicted by various algorithms to affect function (Adzhubei et al. 2010; Ioannidis et al. 2016; Kumar et al. 2009; Schwarz et al. 2014).
  - The 5T/12TG allele (Sosnay et al. 2013).

Variants of unknown clinical significance:
  - Previously reported intronic variants outside of the splice sites with a frequency lower than 1% in the general population and with controversial molecular or clinical data (Casals et al. 2000; Cutting et al. 1992).
  - Previously reported missense variants with contradicting molecular or clinical data in follow‐up studies (Banjar et al. 1999; Bell et al. 2011; Chillon et al. 1994; Dork et al. 1993; Fanen et al. 1992; Lerer et al. 1999; Mickle et al. 1998; Quint et al. 2005; Romey et al. 1994; Schrijver et al. 2005a; Shoshani et al. 1994; Trujillano et al. 2013).
  - Novel silent mutations not creating an amino acid change at the protein level.

Variants with no functional effect:
  - Variants currently defined as non‐CF‐causing mutation by CFTR2 (Cutting et al. 1992; Sosnay et al. 2013).
  - Variants with a frequency higher than 1% in the general population.
  - The 5T/11TG allele (Sosnay et al. 2013).

The molecular findings for each of the patients were discussed with their treating physicians in the context of their clinical, typical, or atypical phenotype. Generally, the molecular findings were compatible with the diagnosis of typical or atypical CF when two mutations that affect function or that probably affect function were identified. The data collected for each of the variants are summarized in Table S2. The mutation c.350G>A; p.Arg177His was specifically searched for by direct observation of the sequences embedding it. Variants with no functional effect are not discussed in this manuscript.

It is important to note that our approach overlaps, but is not identical to the ACMG sequence variant annotation guidelines (Richards et al. 2015). We have deviated from the noted guidelines to account for well‐curated CF‐specific databases such as the CFTR2 database (Sosnay et al. 2013), for the large body of evidence available in the literature for variants not discussed by the same database (Table S2), and for the current Israeli guidelines. Our major concern relates to the lack of clear guidelines for literature survey when assessing the potential pathogenicity of a given variant. Accordingly, the literature survey was not used as a sole evidence for pathogenicity.

## Results

### Clinical and demographic indices

A total of 176 patients participated in this study, 60 of whom belonged to 26 families represented by two to five individuals. Following the completion of the molecular analysis, it became apparent that these 176 patients represent 152 molecular cases based on the combined clinical phenotype and obtained genotypes within each family (Table S3). Further analysis was restricted to the unique molecular cases rather than individuals. Tables 1 and S3 represent the demographic and clinical parameters of the studied population. The 152 cases included 75 (49.3%) cases defined as typical CF and 77 (50.7%) cases defined as atypical CF. A total of 81/152 (53.3%) were of Jewish or mixed Jewish/non‐Jewish ancestry, and 71/152 (46.7%) were of Arab, Bedouin, Druze, or mixed Arab/Caucasian origin.

**Table 1:** Demographic and phenotypic characteristics of the studied cases

*N* mutations	*N* cases	Typical CF						Atypical CF					
		Total	Pre genotyping		Post genotyping			Total	Pre genotyping		Post genotyping		
Ethnicity			One	None	Two	One	None		One	None	Two	One	None
Jews	81	45	32	13	35	5	5	36	22	14	15	9	12
Mixed ancestry	33	22	15	7	17	2	3	11	6	5	4	2	5
Ashkenazi	27	11	10	1	10	1		16	13	3	9	5	2
Ethiopian	5	3		3	1	1	1	2		2		1	1
Libyan	2	2	2		2								
Moroccan	2	1		1			1	1	1		1		
Yemenite	2							2		2			2
Iraqi	1	1		1		1							
Georgian	1							1	1		1		
Uzbek	1							1	1			1	
Jew/non‐Jew	7	5	5		5			2		2			2
Non‐Jews	71	30	13	17	25	2	3	41	7	34	4	7	30
Arab Muslim	56	23	9	14	18	2	3	33	6	27	4	5	24
Druze	7	2	2		2			5		5		1	4
Bedouin	4	3		3	3			1	1			1	
Arab Christian	3	1	1		1			2		2			2
Arab/Caucasian	1	1	1		1								
Total	152	75	45	30	60	7	8	77	29	48	19	16	42

### Identified mutations

We identified 179 variants that affect or probably affect function among the 152 cases that are referred to, herein, as mutations. A total of 54 different mutations (Table 2) were identified, of which 24 mutations were found in two or more cases and 30 were singletons. Of the 54 different mutations, 51 were missense, nonsense, splice site, and small intragenic deletions/insertions mutations. Two mutations were large exonic deletions of exons 19–21 or exons 2–3 and one was the 5T/12TG allele. It was impossible to determine whether the large exonic deletions represent the same molecular event as the break points were not identified. Of the 54 mutations, 29 (53.7%) are currently defined as CF causing by CFTR2, 13 (24.1%) were previously reported to be CF causing in peer‐reviewed manuscripts, 4 (7.4%) are currently defined as mutations of varying clinical consequences by CFTR2, 4 (7.4%) were novel, 3 (5.6%) were previously reported to be causative for CAVD in peer‐reviewed manuscripts, and 1 (1.9%), c.[1075C>A;1079C>A];p.[Gln359Lys;Thr360Lys], is currently defined as a mutation under evaluation by CFTR2. This mutation is included in the Israeli screening panel and was previously suggested to be prevalent among Georgian Jews. Of the 22 mutations currently included in the Israeli preconception screening program, 20 are included in the CFTR2 database (Table S1). Of the 54 mutations found in our dataset only, 16 (29.6%) are currently included in the Israeli preconception screening program (Table 2). The other 38 (70.4%) mutations are not included in the Israeli *CFTR* preconception screening program. Finally, four variants of unknown clinical significance (VOUS) were identified: c.958T>G;p.Leu320Val, c.2620‐15C>G, c.3607A>G;p.Ile1203Val, and c.4242+13A>G (Tables S2 and S4).

**Table 2:** List of all different mutations identified

Mutation^a^	Significance^a^	*N* b	Mutation^a^	Significance^a^	*N* b
c.1521_1523delCTT;p.Phe508del^c^	CFTR2, Causing	34	c.1397C>G;p.Ser466*	CFTR2, Causing	1
c.3846G>A;p.Trp1282*^c^	CFTR2, Causing	24	c.1439G>A;p.Gly480Asp	Reported, CFTR	1
TG12/T5	CFTR2, Varying	24	c.1545_1546delTA; p.Tyr515*fs^c^	CFTR2, Causing	1
c.3276C>A;p.Tyr1092*	CFTR2, Causing	6	c.1585‐1G>A^c^	CFTR2, Causing	1
c.1624G>T;p.Gly542*^c^	CFTR2, Causing	5	c.1736A>G;p.Asp579Gly	CFTR2, Varying	1
c.254G>A;p.Gly85Glu^c^	CFTR2, Causing	5	c.2052dupA; p.Gln685Thrfs*4	CFTR2, Causing	1
c.3472C>T;p.Arg1158*	CFTR2, Causing	5	c.2421A>G; p.Ile807Met	Reported, CAVD	1
c.761delA;p.Lys254Argfs*7	Novel	4	c.2619+1A>G	Novel	1
del exon 19‐21^c^	Reported, CFTR	4	c.2619+2dupT	Reported, CFTR	1
c.3041A>G;p.Tyr1014Cys	Reported, CAVD	3	c.2657+5G>A	CFTR2, Causing	1
c.313delA;p.Ile105Serfs*2	CFTR2, Causing	3	c.273+1G>A^c^	CFTR2, Causing	1
c.3299A>C;p.Gln1100Pro	Reported, CFTR	3	c.2989‐1G>A^c^	CFTR2, Causing	1
c.3454G>C;p.Asp1152His	CFTR2, Varying	3	c.3160C>G;p.His1054Asp	CFTR2, Causing	1
c.3883_3886delATTT;p.Ile1295Phefs*32^c^	CFTR2, Causing	3	c.3196C>T;p.Arg1066Cys	CFTR2, Causing	1
c.3909C>G;p.Asn1303Lys^c^	CFTR2, Causing	3	c.3276C>G;p.Tyr1092*^c^	CFTR2, Causing	1
c.416A>T;p.His139Leu	Reported, CFTR	3	c.3469‐2A>G	Reported, CFTR	1
c.4251delA;p.Glu1418Argfs*14	CFTR2, Causing	3	c.3717+12191C>T^c^	CFTR2, Causing	1
c.[1075C>A;1079C>A];p.[Gln359Lys;Thr360Lys]^c^	CFTR2, Unknown	2	c.3808G>A;p.Asp1270Asn	CFTR2, Varying	1
c.1393‐1G>A	CFTR2, Causing	2	c.3883delA; p.Ile1295Phefs*33	CFTR2, Causing	1
c.1911delG;p.Gln637Hisfs*26	Reported, CFTR	2	c.3889dupT; p.Ser1297Phefs*5	CFTR2, Causing	1
c.2988+1G>A	CFTR2, Causing	2	c.413_415dupTAC; p.Leu138dup	Reported, CAVD	1
c.3764C>A;p.Ser1255*	CFTR2, Causing	2	c.4297G>A;p.Glu1433Lys	Novel	1
c.523A>G;p.Ile175Val	Reported, CFTR	2	c.4364C>G;p.Ser1455*	Reported, CFTR	1
del exon 2‐3^c^	CFTR2, Causing	2	c.487A>G;p.Lys163Glu	Novel	1
c.1000C>T;p.Arg334Trp	CFTR2, Causing	1	c.575A>G;p.Asp192Gly	Reported, CFTR	1
c.1001G>A;p.Arg334Gln	Reported, CFTR	1	c.675T>A;p.Cys225*^c^	Reported, CFTR	1
c.1364C>A;p.Ala455Glu	CFTR2, Causing	1	c.870‐2A>G	Reported, CFTR	1

For the legacy and protein names of the mutations see Table S3.

a CFTR2, Causing = CF causing by CFTR2; CFTR2, Varying = CF varying consequences by CFTR2; CFTR2, Unknown = CF unknown significance by CFTR2; Reported, CFTR = previously reported to be CF causing in a peer‐reviewed manuscript; Reported, CAVD = previously reported to be causative for CAVD in a peer‐reviewed manuscript; Novel = first reported in this study.

b *N* refers to the number of chromosomes in which the mutations were identified.

c Mutations included in the Israeli CF preconception program (Table S1).

Four cases demonstrated complex alleles and were not counted as additional different mutations (Table S3). The mutation c.3209G>A; p.Arg1070Gln reported to be of varying clinical consequences by CFTR2 was identified twice. Once it was identified with c.1521_1523delCTT;p.Phe508del/c.3196C>T;p.Arg1066Cys, and once with c.3846G>A;p.Trp1282*/c.1397C>G;p.Ser466*. All four mutations are considered as CF causing by CFTR2. The mutation c.220C>T; p.Arg74Trp reported to be of varying clinical consequences and the mutation c.601G>A; p.Val201Met predicted to be deleterious were identified with c.1521_1523delCTT;p.Phe508del/c.3808G>A;p.Asp1270Asn. The VOUS c.3607A>G;p.Ile1203Val was identified with c.1521_1523delCTT;p.Phe508del/c.3764C>A;p.Ser1255*. The effect of these mutations or the VOUS on the CFTR protein function when found in a complex allele formation was not further studied. These four cases were regarded as carrying two mutations and molecularly resolved.

### Genotype–phenotype correlation

Table 3 intersects the identified mutation categories and the clinical phenotype. Table S3 contains the mutations identified within each of the groups. Two mutations that affect or probably affect CFTR function were found in 78/152 (51.3%) cases, of which 60/78 (76.9%) were defined clinically as typical CF and 18/78 (23%) as atypical CF. A total of 104/156 (66.7%) chromosomes observed in these cases carried a CF‐causing mutation as defined by CFTR2, making it the largest of the groups.

**Table 3:** Clinical phenotypes and molecular genotypes correlations

CF type	Typical CF	Atypical CF	Total
*Two mutations (allele1/allele2)*	60	18	78
CFTR2, Causative/CFTR2, Causative	35	2	37
CFTR2, Causative/CFTR2, Varying consequences	4	11	15
CFTR2, Causative/Novel	1	0	1
CFTR2, Causative/Previously reported CAVD	3	1	4
CFTR2, Causative/Previously reported CFTR	8	1	9
CFTR2, Causative/CFTR2, Under evaluation	0	1	1
CFTR2, Varying consequences/CFTR2, Varying consequences	1	2	3
Previously reported CFTR/Previously reported CFTR	5	0	5
Previously reported CFTR/Novel	1	0	1
Novel/Novel	2	0	2
*One mutation (allele1/allele2)*	7	16	23
CFTR2, Causative/None	1	9	10
CFTR2, Under evaluation/None	1	0	1
CFTR2, Varying consequences/None	2	6	8
Previously reported CFTR/None	1	1	2
Previously reported CAVD/None	1	0	2
Novel/None	1	0	1
*No mutations (allele1/allele2)*	8	43	51
None/None	8	43	51
Total	75	77	152

Only one mutation was identified in 23/152 (15.2%) cases; of these, 7/23 (30.4%) were clinically defined as typical CF and 16/23 (69.6%) as atypical CF. In 51/152 (33.6%) cases included in this study for which no mutations were identified, 8/51 (15.7%) were clinically defined as typical CF and 43/51 (84.3%) as atypical CF.

### Molecular investigation of typical versus atypical CF cases

A total of 75/152 (49.3%) cases were clinically defined as typical CF (Table 3). In 60/75 (80%) cases, two mutations were identified so that genotyping and clinical data converged into an established molecular diagnosis. In 7/75 (9.3%) cases, only one mutation was found. In 8/75 (10.7%) cases, no mutations were detected. Of the 60 cases presenting two mutations, 35 (58.3%) carried two CF‐causing mutations per CFTR2 (Table 3).

Atypical CF was defined in 77/152 (50.7%) cases. In only 18/77 (23.4%) cases, the genotyping and clinical data converged into an established molecular diagnosis. Only 2/18 (11.1%) of the cases carried two CF‐causing mutations per CFTR2. In 16/77 (20.7%), only one mutation was found, and in 43/77 (55.8%) cases, no mutations were detected (Table 3).

### Assessment of the current Israeli preconception screening program

**Figure 1:**
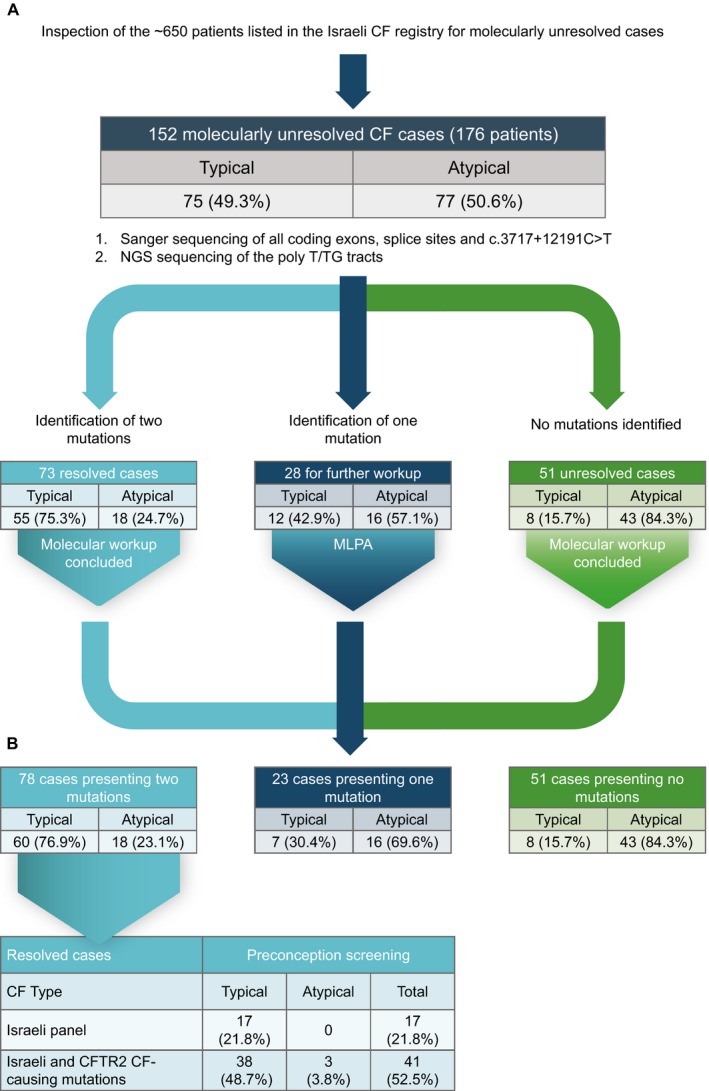
Genotyping strategy. (A) Presentation of the hierarchical genotyping completed on all samples. (B) Theoretical detection rates for preconception screening of CF for the molecularly resolved case. Mutations included in the Israeli panel are listed in Table S1. The theoretical expanded panel for preconception screening comprises the Israeli panel and all CF‐causing mutations by CFTR2.

The Israeli preconception screening program includes 22 ethnically targeted mutations of which four are suggested for specific villages (Table S1). Unexpectedly, the molecular diagnosis of 17/78 (21.8%) cases carrying two mutations could have been established by adhering to the current Israeli guidelines (Fig. 1), and were proven to be noncompliant with the a priori inclusion criteria of the study. The number of cases that could have been diagnosed prenatally by screening for all the mutations included in the Israeli panel and all CF‐causing mutations by CFTR2 is 41/78 (52.5%). CF‐causing mutations included in the CFTR2 database but not in the Israeli program explained 24/78 (30.8%) of the cases. The inclusion of CF mutations with varying consequences to preconception panels, and specifically of c.3454G>C;p.Asp1152His in Israel, has been a source of repeated debate (Peleg et al. 2011; Stafler et al. 2015). Inclusion of these mutations would result in detection of 59/78 (75.6%) of the cases carrying two mutations. Finally, 74/78 (94.9%) of these cases involved previously reported mutations whether included or not included in the CFTR2 database.

### A search for founder mutations

We recorded 14 different ethnicities or mixed ethnicities among all investigated cases (Table S4) and compared the number of mutations found in each population and the number of mutations that would have been covered by the Israeli panel. The panel covered the variations found only among Arab Christians, Libyan Jews, and Uzbeki Jews. In contrast, among Arab Muslims, 14/22 (63.6%) identified mutations, and the two mutations identified among Ethiopian Jews are not included in the standard Israeli panel. These findings suggest that we have not yet reached saturation in some of the sub‐Israeli communities.

When excluding the mutations currently offered in the Israeli panel and the 5T/12TG allele, a total of 36 additional mutations were identified, of which 23 appeared in one case and 13 appeared in multiple cases. The ancestry and kinship for each participant were determined by direct questioning in a medical interview to identify mutations that could represent two or more unrelated cases. Ten mutations fulfilled this criterion which makes them potential candidates for preconception screening for the populations in which they were identified (Table 4). Of the resolved cases, 25/78 (32.1%) involved one of these mutations. It should be noted that the carrier frequencies of the mutations identified in our study were not established which makes it possible that some of the mutations currently noted as singletons might represent additional founder mutations (Table S4). For example, the mutation c.1000C>T;p.Arg334Trp was identified in a compound heterozygosity state in one of our patients. However, inspection of the 2009 Israeli CF registry revealed that only 10/395 (2.5%) patients carrying two mutations presented mutations not included in the Israeli preconception screening program. Evidently, the c.1000C>T;p.Arg334Trp mutation appeared in five of these 10 CF patients, of which, three were homozygotes and two were compound heterozygotes. This suggests that this mutation should be considered as a potential 11th founder mutation.

**Table 4:** List of potential new founder mutations

Mutation	Type	*N* cases	*N* chromosomes (Het/Hom)	Family
c.3472C>T;p.Arg1158*	CFTR2, Causative	5	7 (3/2)	One Arab family, One Bedouin family
c.761delA;p.Lys254Argfs*7	Novel	4	8 (0/4)	Two unrelated Bedouin families
c.3299A>C;p.Gln1100Pro	Previously reported CFTR	4	4 (4/0)	Three unrelated Arab families
c.3276C>A;p.Tyr1092*	CFTR2, Causative	3	6 (0/3)	Three unrelated Arab families
c.4251delA;p.Glu1418Argfs*14	CFTR2, Causative	3	5 (1/2)	Two unrelated Arab families
c.3041A>G;p.Tyr1014Cys	Previously reported CAVD	3	3 (3/0)	Three unrelated Jewish families
c.313delA;p.Ile105Serfs*2	CFTR2, Causative	2	3 (1/1)	One Arab family, One Arab/Jewish family
c.416A>T;p.His139Leu	Previously reported CFTR	2	3 (1/1)	Ethiopian Jews, half siblings
c.2988+1G>A	CFTR2, Causative	2	2 (2/0)	Two unrelated Jewish families
c.3764C>A;p.Ser1255*	CFTR2, Causative	2	2 (2/0)	Two unrelated Arab families
c.1000C>T;p.Arg334Trp	CFTR2, Causative	1	1 (1/0)	One family of mixed Jewish ancestry^a^

a This mutation was identified in five additional patients listed in the Israeli CF data registry.

### Specific considerations

To study for the presence of the same mutations across the various Israeli populations, the ancestry of the populations was collapsed into Jews, Arabs, Druze, and Bedouins. Eight mutations were shared by at least two populations, including c.1521_1523delCTT;p.Phe508del, c.1545_1546delTA;p.Tyr515*fs, c.1624G>T;p.Gly542*, c.3454G>C;p.Asp1152His, c.3472C>T;p.Arg1158*, c.3846G>A;p.Trp1282*, c.3909C>G;p.Asn1303Lys, and del exon 19‐21.

Three combinations of mutations were associated with two different clinical phenotypes. The combination c.1521_1523delCTT;p.Phe508del and T5/TG12 was observed in one patient diagnosed as typical CF and three patients diagnosed as atypical CF. The combination c.254G>A;p.Gly85Glu and c.3472C>T;p.Arg1158* was observed in two patients diagnosed as CF and one patient diagnosed as atypical CF, all from the same village. Homozygosity for the T5/TG12 was observed in one patient diagnosed as typical CF and two patients diagnosed as atypical CF.

The mutation c.3276C>G was identified in one case of mixed Jewish ancestry, while the mutation c.3276C>A was detected in three unrelated families of Arab Muslim origin from the same village. Both mutations produce the same p.Tyr1092* change at the protein level. The mutation c.675T>A;p.Cys225* currently screened in one Arab village was also found in another Arab village. The mutation c.350G>A; p.Arg177His and the 5T/13TG allele were not detected among our patients.

The pathogenic effect of c.4364C>G;p.Ser1455* was repeatedly discussed in the past (Mickle et al. 1998; Salvatore et al. 2005). In our dataset, one typical CF presented a compound state for the mutations c.1521_1523delCTT;p.Phe508del and c.4364C>G;p.Ser1455*. To better understand the role of this variant in CF we extended the genotyping in this family to include two siblings who have not shown signs and symptoms compatible with the diagnosis of CF. Interestingly, both demonstrated the same compound heterozygosity state.

## Discussion

In heterogeneous populations with a wide range of mutations, genetic diagnosis and preconception carrier screening is challenging and many patients may lack a molecular diagnosis. The purpose of our nationwide genotyping project was to find additional CF‐causing mutations in the genetically heterogeneous multiethnic society of Israel. Our intention was that such findings might be beneficial for implementing an expanded, global, pan‐population CF preconception screening panel in Israel. Perhaps the most important and challenging aspect of our study relates to the mere ability to identify the variants existing in the targeted population and infer their potential pathogenic effect (Table 2). This, in turn, holds the largest promise of affecting the number of new mutations to include in an expanded population‐level preconception carrier screening and the prevention of future cases within the general population or the affected families in which molecular diagnosis was lacking. Surprisingly, a total of 17/78 (21.8%) cases presenting two mutations could have been resolved with the current Israeli standard panel (Table S1) and, in principle, should not have been included in our study. The lack of previous molecular diagnosis in these cases might be attributed to screening done by partial historical panels, laboratory errors, or clerical mistakes while documenting laboratory results or collecting patient information. This emphasizes the need for physicians to encourage patients to complete genetic testing and to repeat the genetic testing in cases of high clinical suspicion of CF when no supporting molecular findings are available.

The pathogenicity of the observed variants varied significantly and ranged from mutations accepted to be CF causing in the appropriate molecular context to variants of uncertain clinical significance (VOUS) (Sosnay et al. 2013). There was no debate regarding mutations recognized by CFTR2 to be CF causing or of varying consequences in the appropriate clinical context. Other variants were considered likely to be pathogenic when the observed sequence change is expected to clearly distort the protein structure, when previous peer‐reviewed papers have documented the mutation, or in cases where a novel missense mutation was predicted to be pathogenic by in silico predictive algorithms, including the recently introduced ensemble method, REVEL (Table S2) (Adzhubei et al. 2010; Ioannidis et al. 2016; Kumar et al. 2009; Schwarz et al. 2014). It is clear that final confirmation of pathogenicity mandates functional proof (Sosnay et al. 2013), which remains the focus of future studies. Therefore, it is possible that pathogenic effect was mistakenly attributed to some of the variants and some cases considered to be resolved are not. It should also be noted that we could not verify whether mutations were in *cis* or *trans* configuration. Conversely, VOUS were regarded as of no pathogenic effect until proven otherwise, and therefore, it is possible that several additional cases could be molecularly diagnosed based on the existing results.

Comprehensive preconception screening programs, including that of the *CFTR* gene, have recently been a source of intense discussion (Edwards et al. 2015; Grody et al. 2013; Haque et al. 2016; van der Hout et al. 2016). Screening and diagnostic panels are often requested based on detection rates and residual risks which depend on the tested mutations and the tested population (American College of Obstetricians and Gynecologists Committee on Genetics, 2011; Lim et al. 2016; Richards et al. 2002). Our data cannot accurately set the global detection rate for CF in Israel as it focused strictly on the 152 molecularly unresolved cases, representing 176 patients, of the ~650 known CF patients. However, using the Israeli CF registry data it was suggested that the detection rate of the current Israeli preconception panel (Table S1) is 70% (Stafler et al. 2015). Assuming that the patients included in our study represent the remaining 30% and that molecular diagnosis was reached in 78/152 (51.3%) cases, or 96 patients, some estimates can be made. If all mutations identified in this study are considered, an Israeli pan‐population detection rate of approximately 85% could be reached. Strikingly, it is also clear that the mere inclusion of all mutations appearing in the CFTR2 database without any a priori knowledge of their existence or absence in the Israeli population could have theoretically increased the detection rate in our sample set from 21.8% to 52.5% for preconception screening (Fig. 1). Only four (5.1%) of the resolved cases carried novel mutations not reported elsewhere (Table 2). While the increase in the detection rate for the entire Israeli population under the theoretical inclusion of all CFTR2 mutations is expected to be significant, the effect on the residual risk is shown to be dramatic. Based on our data, it can be cautiously suggested that ethnically indifferent expanded panels are likely to achieve higher detection rates. More specifically, we identified a total of 11 mutations appearing in two or more unrelated cases, of which six are already defined as CF causing by the CFTR2 database, two were previously reported to be CF causing, one was CAVD causing in peer‐reviewed articles, and one was a novel frameshift mutation.

One additional relevant question with respect to preconception carrier screening is related to the appropriate recommendations for a spouse of a carrier. Our data did not address this question directly. However, it is evident that out of the 54 detected mutations in this study only 16 could have been prenatally diagnosed by the current Israeli preconception CF program. The recommendation for an expanded screening panel or of *CFTR* gene sequencing for spouses of carriers should be further considered when more empirical data are available.

The relevancy of our results must be discussed in the context of the genomic era (Larson et al. 2016; Lim et al. 2016; Sosnay et al. 2016). The use of next‐generation sequencing (NGS) for clinical practice is becoming more common. This makes a *CFTR* gene preconception screening test at the level of the whole gene feasible. The use of an NGS platform for such purposes might allow the identification of point mutations, large intragenic deletions/duplications, and assessment of the polyT/TG allele in one test. However, it is important to note that currently we do not recommend the routine use of whole gene sequencing as a method for population‐level screening. Such an approach might yield the unwarranted findings of VOUS or mutations with varying clinical consequences, and is still cumbersome and costly (Edwards et al. 2015). Accordingly, ethnic‐indifferent expanded carrier screening panels based on well‐curated datasets might allow the benefits of much higher detection rates while avoiding the risks associated with whole gene sequencing.

Cystic fibrosis disease expression varies dramatically with respect to the affected organs and the severity of the signs and symptoms (De Boeck et al. 2006; Wallis 2012). It is important to emphasize that the clinical diagnosis of the participants was set at the inception of the project and was not influenced by the molecular results. Detailed investigation of each identified mutation or combination of two mutations in the context of the phenotype presented by each of the patients is beyond the scope of this manuscript. The cases included in this study were those lacking an established molecular diagnosis based on the commonly checked mutations in the Israeli population, therefore it was a priori expected that the fraction of cases representing the more elusive atypical phenotypes would be higher. We dissected our data twice – once from the molecular results and once from the clinical phenotypes. With respect to the genotyping campaign, the cases were divided into those presenting two mutations, one mutation, or no mutations. The clinical and molecular indices of these groups differed significantly. Among the 78 cases carrying two mutations, 60 (76.9%) were diagnosed as typical CF (Table 3). Conversely, the fraction of atypical CF was the largest among the group of cases carrying one (69.5%) or no mutations (84.3%). With respect to the phenotype, the studied cases were classified as typical or atypical CF cases. The molecular results concurred with the clinical phenotype. While among typical CF cases a molecular diagnosis could have been reached in 80%, it was possible only in 23.3% of the atypical CF cases. Moreover, the profile of the mutations identified in the different groups varies significantly. While among typical CF cases the number of chromosomes carrying a CF‐causing mutation by the CFTR2 database was 58%, it was only 17.5% among atypical CF cases. Strikingly, among the 60 typical CF cases carrying two mutations, only five (8.3%) involved a CF‐varying consequences mutation by the CFTR2 database. Among the 18 atypical CF cases carrying two mutations, 13 (72.2%) presented such a mutation. Nevertheless, no matter how different the molecular characteristics of the various groups are, the overall carrier frequency of one mutation in any of the clinical groups is much higher than the expected carrier rate in the general population (Abeliovich et al. 1992; Richards et al. 2002; Zlotogora et al. 2016). It is possible that under certain, yet unknown molecular circumstances involving intragenic, gene–gene, gene–environment, or epigenetic factors, the carrier state for one mutation might have some clinical effect. Such an effect of a single mutation is well‐known to be associated with CAVD (Casals et al. 2000).

It was previously argued that *CFTR* sequence analysis, including all the coding sequences, splice donor, and acceptor sites, the promotor region, and two intronic sequences yields a detection rate exceeding 98% (Strom et al. 2003). Based on this argument, the fact that in 74 (48.7%) of 152 cases a firm molecular diagnosis was not achieved needs to be explained. First, it should be emphasized that this lower detection rate refers only to the portion of the Israeli CF registry patients that had no molecular diagnosis prior to this study. As discussed, for the total of ~650 CF patients in Israel the detection rate following this study might reach 85%. Second, it was previously shown that molecular resolution cannot be achieved in many atypical CF patients using a similar molecular approach (Groman et al. 2005). Third, it should be further emphasized that atypical patients presenting no mutations might be misdiagnosed and actually represent a spectrum of other diseases with signs and symptoms overlapping or mimicking CF (Groman et al. 2002; Sheridan et al. 2005). Fourth, it is possible that some of the patients have mutations in genomic locations that were not screened in this study such as the promotor or deep intronic regions (Strom et al. 2003). Considering all the above, we have recalculated the detection rate among typical Israeli CF patients. A total of ~500/650 of the CF patients are diagnosed as typical CF cases. Following this study, we are aware of only 15 typical CF patients remaining with no molecular diagnosis compatible with CF. This, therefore, indicates a detection rate of ~97% (485/500) for typical CF patients which is compatible with previous reports (Strom et al. 2003). Finally, the possibility of sample mix‐up and of laboratory‐related errors must be considered. Accordingly, the cases not presenting two mutations should be further discussed with respect to their clinical phenotype. Naturally, the seven and eight typical CF cases presenting one or no mutations, respectively, should be the leading candidates for resampling, regenotyping, and for extended genotyping work‐up at the level of the exome or whole genome in an attempt to decipher the molecular reason explaining their clinical manifestations.

In summary, the nationwide genotyping campaign described herein resulted in molecular resolution for 78 cases (96 patients) who will now benefit from better genetic counseling. Changing demographics expressed by the loss of affiliation to one traditional community or geographic location and the clear occurrence of many different mutations across various communities provides motivation for a uniform pan‐population screening program for CF. The identification of 38 mutations that were not known to be part of the mutations affecting the Israeli population, and the understanding of the potential benefit of expanded preconception screening using a well‐curated list of CF‐causing mutations could lead to a revision of the *CFTR* screening panel currently suggested in Israel. Functional studies on all identified mutations are ongoing to supply final proof for their pathogenic role and disease consequences. Finally, it should be noted that molecular resolution is still lacking for about 15% of the Israeli CF registry patients for whom genetic investigation continues.

## Conflict of Interest

The laboratory work presented in this study was conducted by Gene by Gene, Ltd., in which D.M.B and C.B. declare stock ownership, and for which G.A. serves as a consultant. O.I. is the chairman of The Cystic Fibrosis Foundation of Israel. The other authors declare no conflict of interest.

## Supporting information


**Table S1.** List of mutations comprising the Israeli CF preconception program.

**Table S2.** Suggested pathogenicity of all mutations not listed in the CFTR2 database.

**Table S3.** Detailed genotyping results of all participating patients.

**Table S4.** List of all different mutations identified per ethnicity.
